# The healing effect of the collagen-glycosaminoglycan copolymer on corneal thinning

**DOI:** 10.1186/s12886-018-0947-3

**Published:** 2018-10-25

**Authors:** Shu-Ya Wu, Chien-Yi Pan, Elizabeth P. Shen, I-Shiang Tzeng, Wei-Cherng Hsu

**Affiliations:** 1 Department of Ophthalmology, Taipei Tzu Chi Hospital, Buddhist Tzu Chi Medical Foundation, No. 289, Jianguo Rd., Xindian Dist., New Taipei City 231, Taipei, Taiwan (R.O.C.); 2 Wu Chou Animal Hospital, Taipei, Taiwan; 3 Department of Research, Taipei Tzu Chi Hospital, Buddhist Tzu Chi Medical Foundation, Taipei, Taiwan; 4 Tzu Chi University College of Medicine, Hualien, Taiwan

**Keywords:** 3D scaffold, Collagen-glycosaminoglycan copolymer, Corneal thinning, Healing process

## Abstract

**Background:**

To study the healing processes of partial thickness wounds in the adult rabbit cornea after grafting a porous collagen-glycosaminoglycan copolymer matrix (CG).

**Methods:**

In this study, the regeneration of surgically-induced rabbit corneal defect implanted with CG was investigated. The corneal partial thickness wound was created by 7.5 mm trephine. The wound was implanted with CG. Effects on wound healing was analyzed using clinical data on epithelial migration and corneal thickness, and histological data on collagen and alpha smooth muscle actin distribution.

**Results:**

Compared with control group, CG induced a relatively severe inflammatory reaction in grafted cornea until the CG matrix was completely degraded. The new vessel ingrowth and stromal regeneration maintained the corneal thickness. The grafted cornea was significantly thicker (*P* < 0.001) than the control group. On day 90, the corneal opacity score of the control group was one and the grafted cornea was two.

**Conclusion:**

CG copolymer matrix can successfully repair the damaged corneal stroma by injury, and regain its thickness. However, CG matrix induced inflammatory healing process thus causing mild corneal haziness and neovascularization.

## Background

Corneal melting with perforation is a severe, vision-threatening complication of corneal disorders such as corneal ulceration, chemical burn and autoimmune keratitis. In the acute stage, the urgent approach is to limit inflammation by directing against the cause as well as to optimize epithelial healing [1]. Furthermore, surgical procedures, including tissue adhesive [1, 2], amniotic membrane transplantation [1, 3], conjunctival flaps [1, 3], pericardial membrane graft [4], are done to temporarily maintain the integrity of the globe. In general, this involves a multistage surgery [1]. The final tectonic keratoplasty is performed to restore visual function. The surgical methods include full-thickness penetrating keratoplasty, lamellar corneal patch graft, and deep anterior lamellar keratoplasty [1, 5–8], depending on the size and location of perforation. In these procedures, it is essential to have graft material for repair of the corneal defect readily available.

Shortage of graft material for repair, particularly in developing countries, has prompted the development of bioengineered tissue alternatives. Tissue engineering relies on the use of three-dimensional porous scaffolds to provide appropriate microenvironment to induce regeneration of injured tissues and organs. The porous collagen-glycosaminoglycan copolymer matrix (CG matrix) is composed of type I collagen and chondroitin 6-sulfate. The collagen of the corneal stroma is largely type I collagen. Previous study demonstrated that CG matrix in eyes could modulate the healing procedure of conjunctival wound, reducing scarring contraction and promoting the formation of a near-normal subconjunctival stroma [9]. The CG matrix also serves as a three-dimensional scaffold for cell migration and proliferation on surgical bleb defect to maintain the size of bleb and repair the leakage [10, 11]. CG matrix supplies good biocompatibility on the ocular surface. Therefore, CG matrix has a potential to be used as an alternative graft material for repair of corneal thinning by suppling thicker extracelluar matrix in the wound bed. However, considering that diameter, spacing, and spatial orientation of the collage fibrils in the corneal stroma is essential for corneal transparency [12], we tested the healing effect of CG matrix on corneal thinning in a rabbit model. This study provides preliminary results for further advanced studies.

## Methods

### Animals and model of corneal thinning

**Fig. 1:**
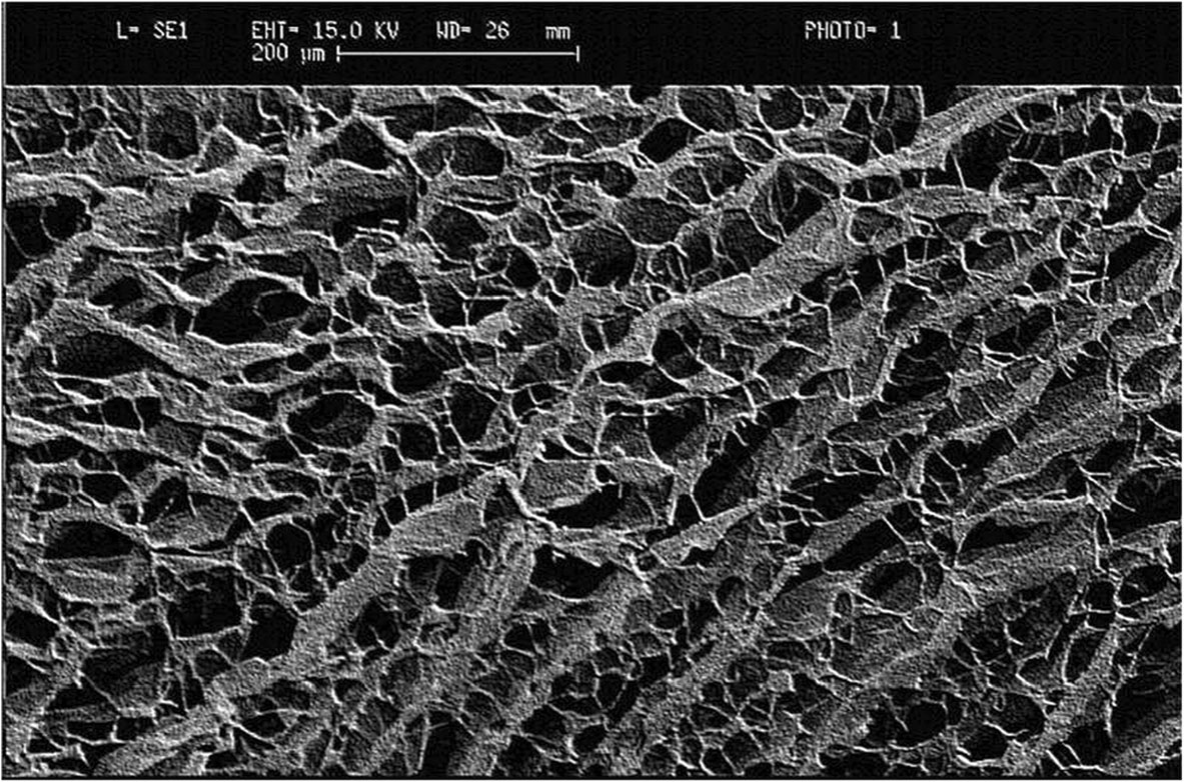
Scanning electron micrograph of the collagen-glycosaminoglycan copolymer matrix implant

All investigations conformed to the ARVO statement for the use of Animals in Ophthalmic and Vision Research. A drug-free, biodegradable, porous collagen matrix of 1% collagen/C-6-S copolymer (iGen) measuring 7.5 mm in diameter and 2 mm in thickness was used. The synthesis of the CG matrix was described in previous study but the type I collagen was purified from porcine skin [10]. The pores of the matrix ranged from 20 μm to 200 μm (Fig. 1). Twenty-four female New Zealand albino rabbits (Level Biotechnology Inc.) weighing 2.5–3.5 Kg were anesthetized by intramuscular injection of ketamine (35 mg/kg) and xylazine (5 mg/kg). Surgical procedures were done under surgical microscope with the eyelids held open by a spectrum. Both eyes underwent deep lamellar keratectomy (DLK) with 7.5 mm trephine to a depth of almost 1/2 the corneal thickness without damaging the corneal endothelium. The right eye served as control and was not implanted with CG matrix (ungrafted eye); while the left eye, as study group, had CG matrix laid over the cornea bed (grafted eye). Both eyes were then covered with therapeutic soft contact lens (Purevision, Bausch & Lomb). Lateral tarsorrhaphy was performed over one-third length of the eyelids to prevent loss of contact lens.

Healing effects of CG matrix on the corneal thinning were assessed on days 3, 7, 14, 28, 60, and 90 after surgical procedures (assessment days). Animals were euthanized by intracardinal injection of sodium pentobarbital (398 mg/ml at 1.0 ml/10 lb. body weight). Immediately after euthanising, eyes were enucleated from the orbit.

### Slit-lamp examination and clinical score of corneal opacity

Slit lamp examination was performed to evaluate corneal opacity on assessment days. Corneal opacity was scored using a five-point scale where 0 is no opacity, completely clear cornea; 1 is sight haziness, iris and lens visible; 2 is moderately opaque, iris and lens still detectable; 3 is severely opaque, iris and lens hardly visible; and 4 is completely opaque, with no view of iris and lens [13].

### Corneal thickness measurements

In all rabbits, corneal thickness of both eyes was measured by Schiempflug photography before and after DLK on surgery day, and on assessment days. Wilcoxon signed-rank test (*P* < 0.001) was used to compare corneal thickness between pre- and post-operation, as well as between control and study eyes over time.

### Histopathologic and immune staining studies

Four rabbits were euthanized on each assessment day. Close upper and lower eyelid sutures were performed to protect the cornea. Entire eye globes were harvested from the orbit and were soaked in Modified Davison’s fluid for 24 h. The globes were fixed in 10% neutral buffered formalin. Corneas with limbus were harvested and embedded in paraffin. The 7 μm sectioned corneas were stained with Masson’s trichrome to evaluate the distribution of collagen. With Masson’s trichrome stain, collagen was stained blue, cellular material stained red, and cell nuclei stained purple. Additional tissue sections were used for alpha-smooth muscle actin (α-SMA) immunocytochemistry to identify myofibroblasts [14–16].

## Results

### Day 3

**Fig. 2:**
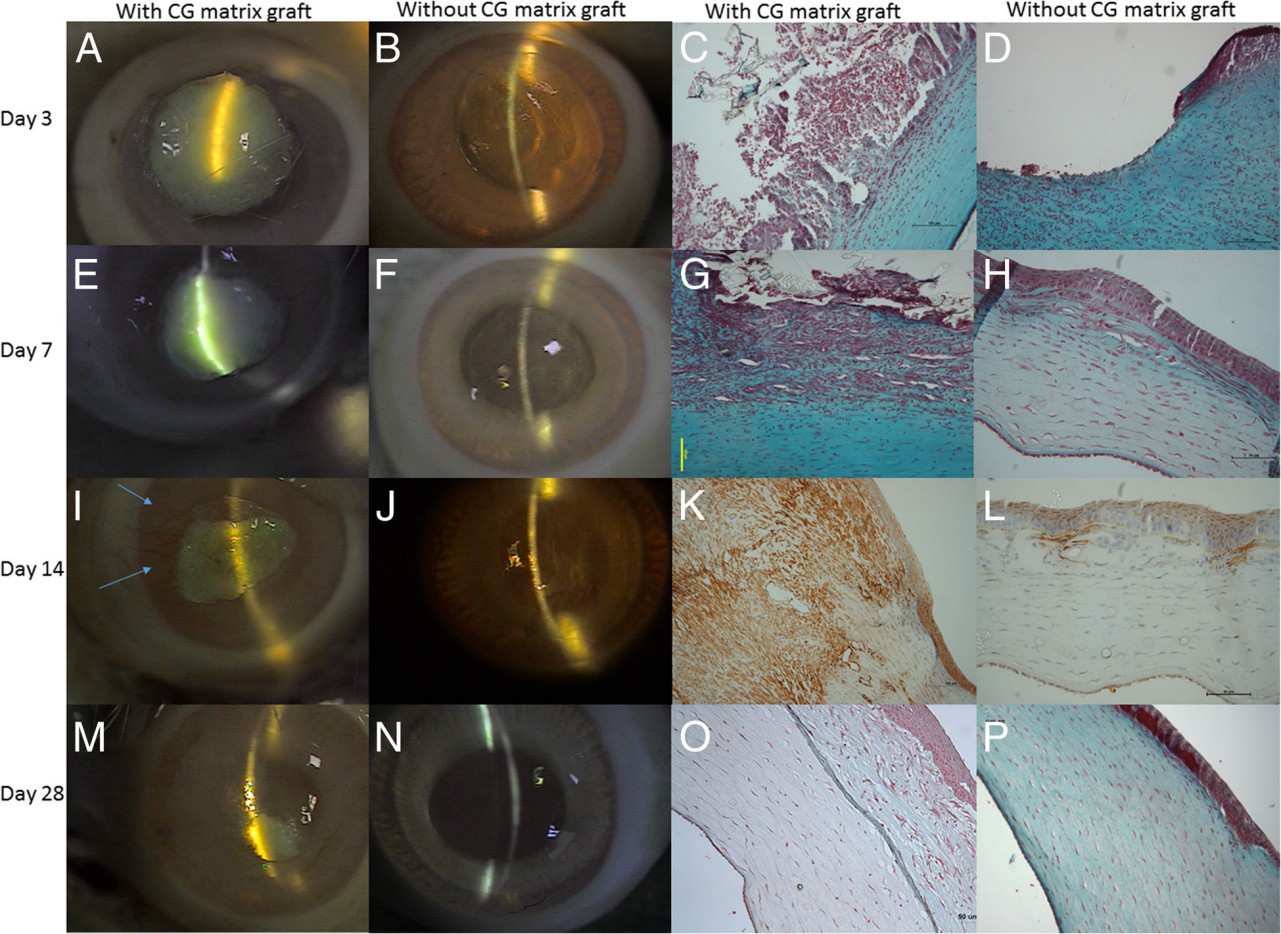
Photographs of slit-lamp examination and histopathological results on day 3, 7, 14, 28 after surgery. **a** Matrix- grafted cornea (**b**) Ungrafted cornea. Masson’s trichrome stain showing (**c**) massive inflammatory cells infiltration around the corneal wound with CG matrix and collagen ingrowth (blue stain). **d** only some inflammatory cell infiltration and re-epithelialization. **e** Matrix- grafted cornea with tiny vessel ingrowth (**f**) Ungrafted cornea wound margin identified by the blue arrows with edema. Masson’s trichrome stain showing (**g**) predominantly fibroblasts, epithelial cells and inflammatory cells into CG matrix with collagen regeneration. **h** epithelial and stromal regeneration (100×). **i** Matrix-grafted cornea with new vessels (blue arrow) and CG matrix degradation over upper corneal wound. **j** Ungrafted cornea with reduced wound area. α-smooth muscle actin stain showing (**k**) strong positive cells over regenerative corneal stroma. **l** only some positive cells infiltrated into the regenerative stroma of the ungrafted cornea (100×). **m** CG matrix-grafted cornea with incomplete degradation and decreasing neovascularization. **n** Ungrafted cornea with near complete healing. One corneal opacity score. Masson’s trichrome stain showing (**o**) irregular arrangement of new collagen deposition upon complete degradation of collagen-GAG scaffold wound with thickening the stroma thickness. **p** complete re-epithelization and wound healing

On post-DLK day 3, the ungrafted eyes (right eyes) showed moderate corneal opacity with iris and lens detectable (corneal opacity score of 2, Fig. 2b); while corneas of grafted eyes (left eyes) were completely opaque (corneal opacity score of 4) due to CG matrix graft (Fig. 2a). Silt-lamp examinations of grafted eyes showed central cornea to be thicker than the peripheral cornea (Fig. 2a).

Histopathologic study with Masson’s trichrome stain revealed acute inflammation in both groups (Fig. 2c and d). In the grafted eyes, polymorphonuclear neutrophils (PMNs), epithelial cells, and fibroblasts were found around and adherent to the CG matrix implant. In the ungrafted eyes, only PMNs and re-epithelial cells migrated and adhered to the corneal wound. The staining for α-SMA was negative in both groups.

### Day 7

On post-DLK day 7, the cornea of the ungrafted eyes became more opaque (corneal opacity score of 3) because of corneal edema (Fig. 2f); while the corneal opacity of the grafted eyes remained at 4 due to incomplete CG matrix degradation (Fig. 2e). With regard to histopathologic findings, the grafted eyes showed marked re-epithelial cells infiltration, and prominent deposition of collagen and fibroblast-like cells in line with stromal regeneration of corneal wound (Fig. 2g). In the ungrafted eyes, stromal regeneration and re-epithelialization were found to a lesser degree (Fig. 2h). The intensity of α-SMA staining was stronger in grafted eyes than in ungrafted eyes.

### Day 14

Through slit-lamp examination, the ungrafted eyes showed clearer corneas (corneal opacity score of 2) and the re-epithelization of corneal wounds was complete (without positive Fluorescein stain) (Fig. 2j). In contrast, the corneas of grafted eyes were still opaque due to presence of CG matrix, the corneal opacity score remained at 4 (Fig. 2i); however, the periphery were healing, clearer, and had new vessels.

In the ungrafted eyes, corneal wound displayed lesser inflammatory reaction and presence of some cells positively stained with α-smooth muscle actin stain (Fig. 2l). The corneas of grafted eyes had comparatively thicker epithelial layer and strong positive cells over regenerative stroma on α-smooth muscle actin stain (Fig. 2k).

### Day 28

In the ungrafted eyes, the corneal opacity score was 1 (Fig. 2n). Histologically, the wound healing process of the ungrafted eyes was deemed complete by negative α-smooth muscle actin stain and absence of inflammatory cells (Fig. 2p).

**Fig. 3:**
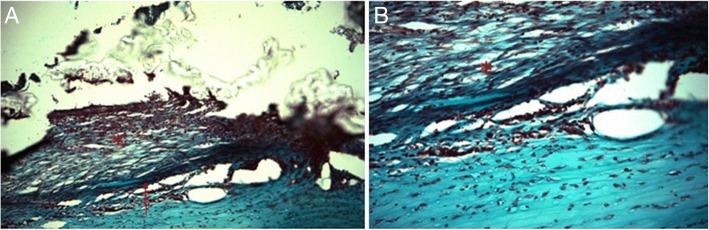
Micrographs at two different magnification powers (**a**) 100× (**b**) 200× of CG matrix grafted cornea on day 28. Masson’s trichrome stain showing (**a**) high cellularity and irregular collagen deposition (red star) upon corneal wound bed (red arrow) with incomplete collagen-GAG scaffold degradation (**b**) CG matrix maintaining the porous scaffold (red star)

In the grafted eyes, CG matrix was still incompletely degraded, thus the corneal opacity score remained at 4 but the peripheral areas, without CG matrix, had score of 3 (Fig. 2m). Compared to post-DLK day 14, the number and diameter of new vessels decreased (Fig. 2m); and loosely organized collagen fibers and fibroblasts-like cells surrounded the partially degraded CG matrix (Fig. 3a and b).

### Days 60 & 90

**Fig. 4:**
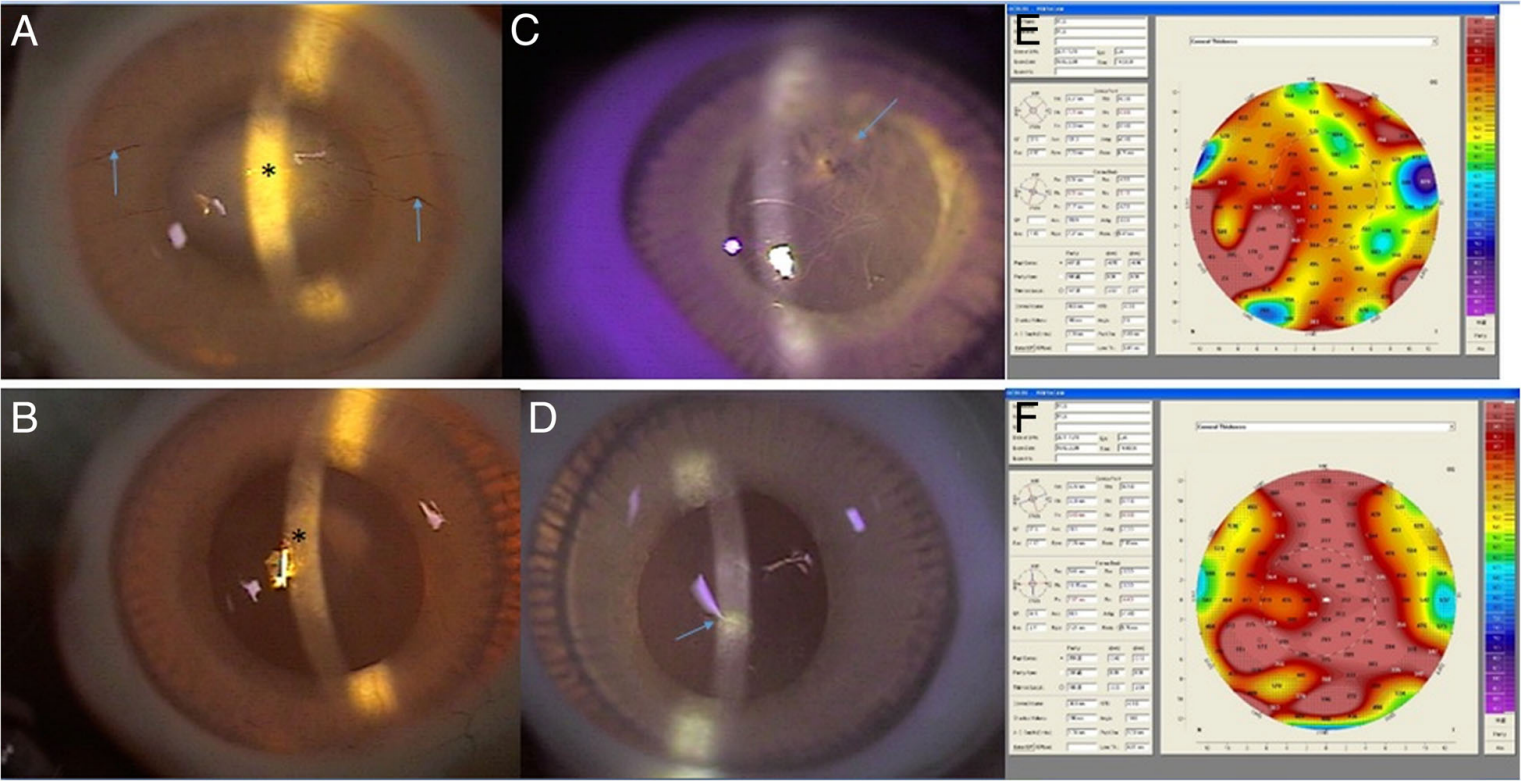
Postoperative day 60, slit-lamp examination revealing (**a**) central scar formation (black star) with vessel ingrowth on matrix-grafted cornea (blue arrow), and complete CG matrix degradation (**b**) more transparent central cornea in control eye. Postoperative day 90, slit-lamp examination showing (**c**) the transparency of matrix-grafted cornea being more limpid (black star) than (**a**) the cornea on day 60, but less transparent than the ungrafted cornea (**d**). Scanning Pentacam photographs on day 90 revealing the thickness of CG matrix-grafted cornea (**e**) being thicker than the control one (**f**). The areas indicated by the arrows in Figs. **e** and (**c**) show consistent results by two different methods. The same as for Figs. (**f**) and (**d**)

On post-DLK days 60 and 90, the corneas of ungrafted eyes remained clear (corneal opacity score maintained at 1) (Fig. 4b and d). On post-DLK day 60, the corneas of the grafted eyes had corneal opacity score of 3 because of scar formation though the CG matrix was completely degraded (Fig. 4a). By post-DLK day 90, corneas of grafted eyes became clearer and the score was 2 (Fig. 4c).

**Fig. 5:**
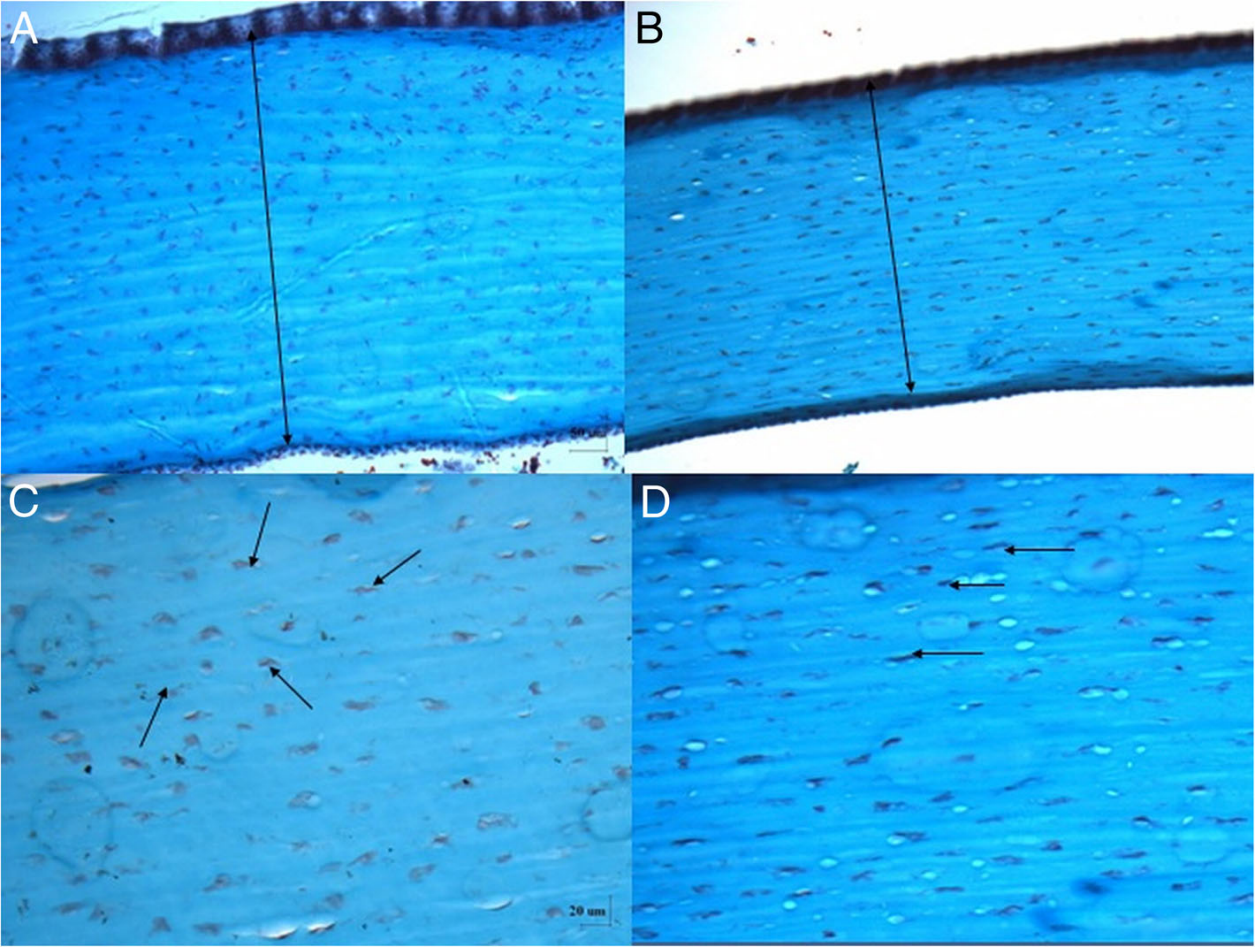
The corneal thickness of collagen-GAG scaffold (**a**) is thicker than the control one (**b**) as indicated by the arrows (Masson’s Trichrome stain, 100×). The arrangement of keratocytes (black arrows) in collagen-GAG scaffold treated wound are more random (**c**) than control one (**d**) as indicated by the black arrows (Masson’s Trichrome stain, 200×)

On post-DLK day 90, cornea of grafted eyes were obviously thicker than those of ungrafted eyes (Fig. 4e and f) but the arrangement of keratocytes showed a more randomized pattern over a superficial layer of stroma (Fig. 5).

**Fig. 6:**
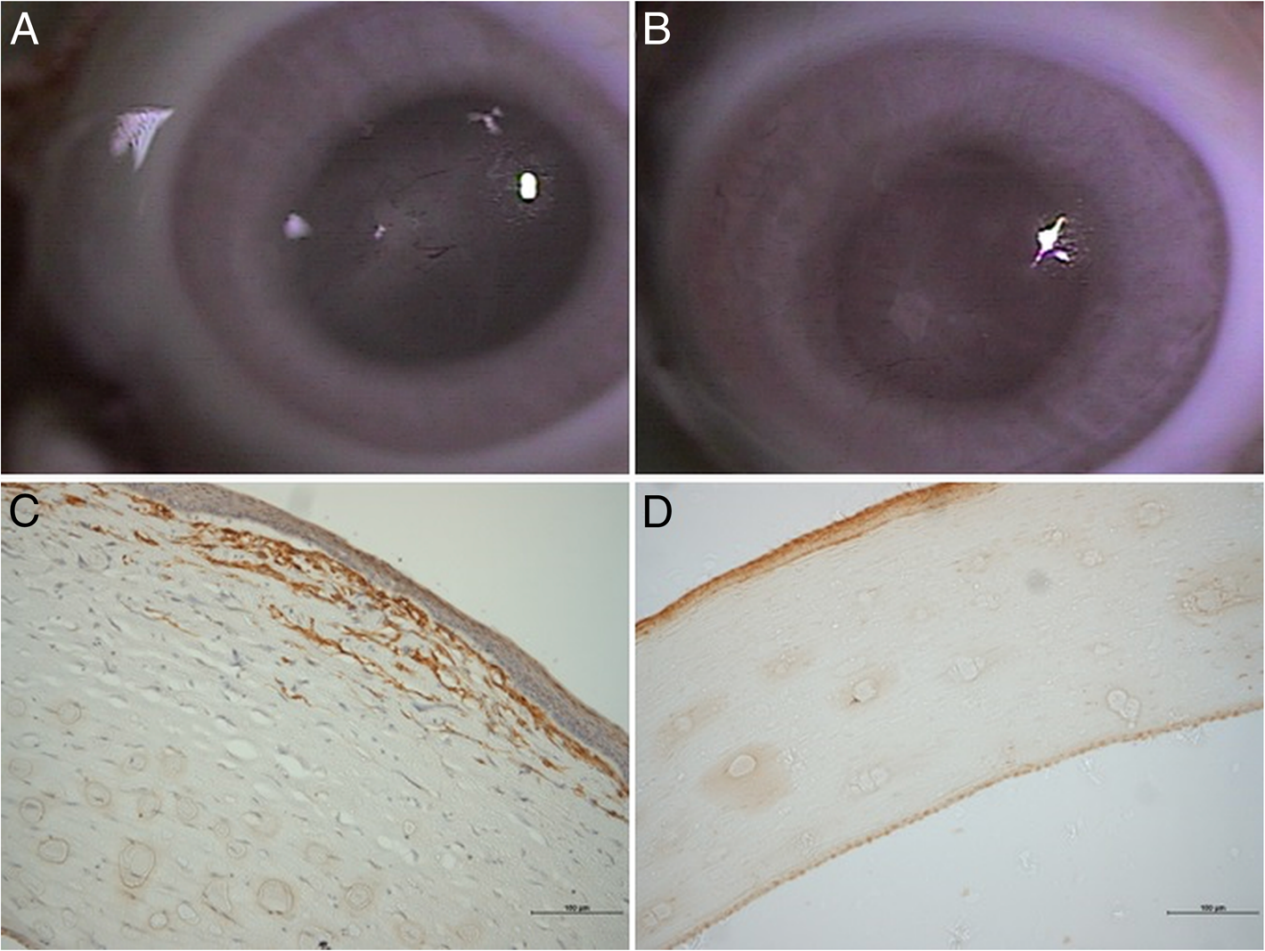
External eye photographs on day 60 of (**a**) CG matrix-grafted cornea and (**b**) ungrafted cornea.α-SMA stains showing (**c**) positive cells found in the stroma of matrix-grafted cornea and (**d**) no positive cells in ungrafted cornea (100×)

On post-DLK day 60, grafted eyes showed presence of positively α-SMA stained cells in the stroma of the corneal wound without porous structure. There were no positively α-SMA stained cells in the ungrafted eyes (Fig. 6).

### Corneal thickness measurements

Corneal thickness of the ungrafted eyes (right eyes) before DLK (mean ± SD) was 317.92 ± 20.33 μm and 158.29 ± 27.30 μm after DLK, while that of the grafted eyes were 317.44 ± 21.04 μm and 151.3 ± 31.51 μm, respectively. The difference in corneal thickness between pre-operation and post-operation was statistically significant (*P* < 0.001) in both the ungrafted and grafted eyes. Between grafted and ungrafted eyes, the post-DLK corneal thickness was not statistically significant.

**Fig. 7:**
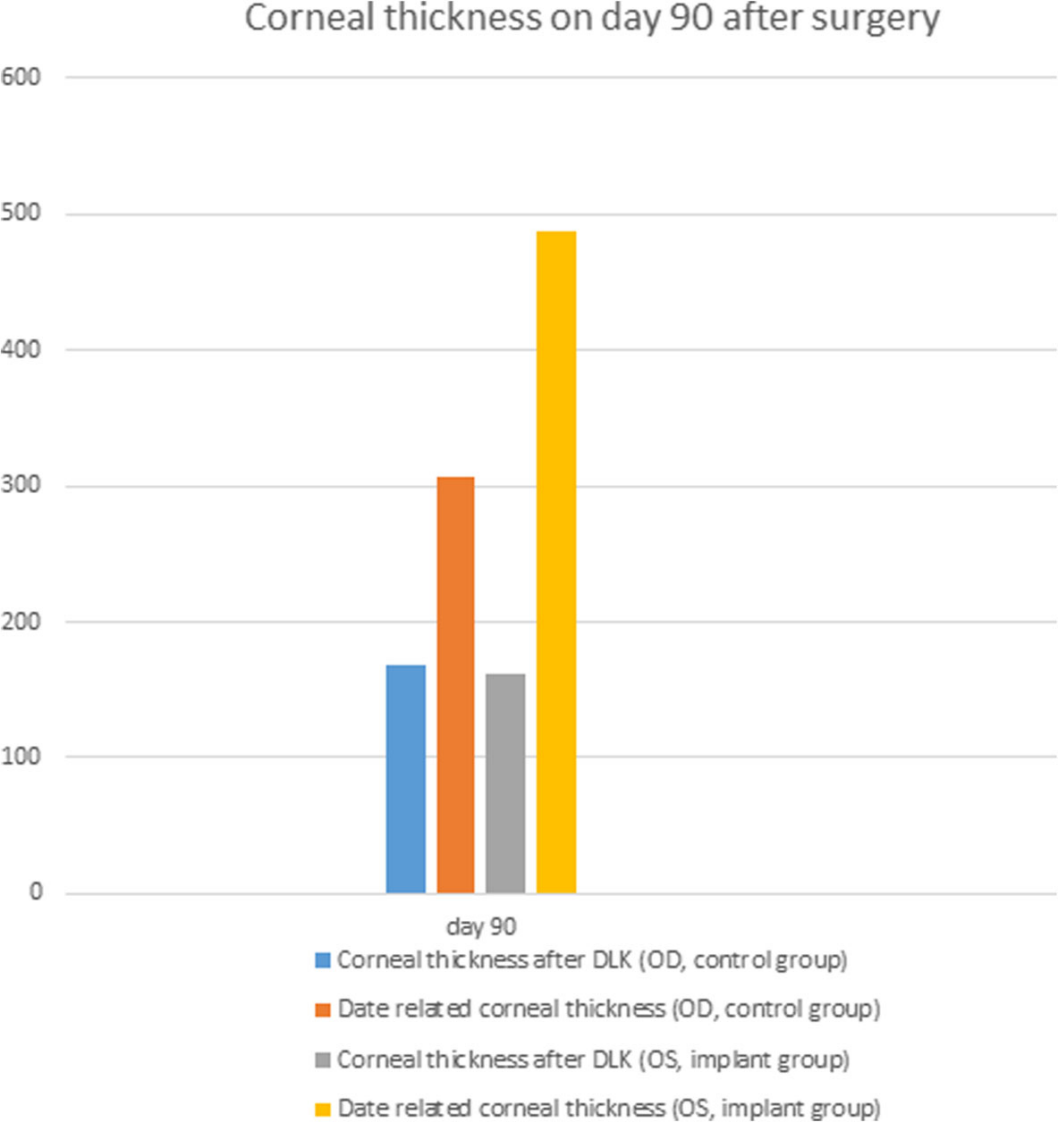
Comparison of corneal thickness on day 90 after surgery

The mean corneal thickness of the ungrafted eyes were 245 ± 15.59 μm, 274.5 ± 15.62 μm, 315.5 ± 6.29 μm, 311.75 ± 7.50 μm, and 307.25 ± 11.19 μm on post-DLK days 3, 7, 28, 60, and 90, respectively. The corneal thickness on post-DLK day 14 was not measured due to corneal edema. For the grafted eyes, mean central corneal thickness was only measurable by pentacam on post-DLK day 90 at 487.25 ± 14.89 μm; on the other prior assessment days, the pentacam failed to measure the central corneal thickness due to presence of non-degraded CG matrix and opacity. The mean central corneal thickness of grafted eyes was significantly thicker (*P* < 0.001) than the ungrafted eyes on post-DLK day 90 (Fig. 7). The results and comparisons between grafted and ungrafted eyes were summarized in Table 1.

**Table 1:** The summary of the results and comparison between collagen-glycosaminoglycan copolymer matrix grafted cornea and un-grafted cornea

	Day 3	Day 7	Day 14	Day 28	Day 60	Day 90
Corneal opacity score						
grafted cornea	4	4	4	4	3	2
un-grafted cornea	2	3	2	1	1	1
Corneal thickness						
grafted cornea(μm)	NA	NA	NA	NA	NA	487.25 ± 14.89
un-grafted cornea(μm)	245 ± 15.59	274.5 ± 15.62	NA	315.5 ± 6.29	311.75 ± 7.50	307.25 ± 11.19
Neovascularization						
grafted cornea	–	+	+	+	+	+
un-grafted cornea	–	–	–	–	–	–
α-SMA-positive cell						
grafted cornea	–	+	+	+	+	–
un-grafted cornea	–	+	+	–	–	–

*NA* not applicable, + positive, − negative

## Discussion

Corneal thinning by trauma, surgery, infection, or inflammation triggers a series of corneal wound healing processes and corneal matrix remodeling, including stromal keratocyte apoptosis, epithelial migration, myofibroblast proliferation, and fibrosis [17, 27, 28]. However, these responses might lead to angiogenesis and compromise the restoration of corneal transparency [18, 19]. In addition, if incomplete wound healing persists, it could cause no improvement of corneal thickness and even perforation [20]. Currently, the ultimate management to correct stromal scarring and corneal thinning is corneal transplantation. Full or partial-thickness corneal grafts are effective means of restoring transparency and thickness, but this procedure relies on fresh donated cadaveric cornea. Due to shortage of donor cornea, it is imperative to find viable alternatives to corneal tissue.

Type I collagen accounts for about 85% of the fibrillar collagen in human corneal stroma. In the form of heterotypic fibrils with type V collagen, type I collagen are crucial for corneal transparency [21]. The porous collagen-glycosaminoglycan copolymer matrix (type I collagen and chondroitin 6-sulfate) was designed as temporary scaffolds with stiffness that maintains corneal structure in corneal thinning, while the surrounding corneal stroma tissue regenerates and replaces the original scaffold over time. In this study, CG matrices were implanted in the corneal stroma of 24 rabbits after deep lamellar keratectomy and observed over a 90-day period thereafter. On day 3, the grafted cornea showed infiltration of acute inflammatory cells around CG matrix. On day 7, new vessels began to appear at the periphery of the corneal wound. The CG matrix degraded completely between days 28 and 60. On day 60, CG matrix disappeared and central corneal scar formation with peripheral vessels ingrowth were found. And on day 90, the cornea of the grafted eyes cleared (Table 1). From the histopathologic findings, the intensity of α-SMA staining increased progressively from 7th to the 14th day then decreased gradually overtime but remaining positive at day 60. Therefore, CG matrix induced stronger inflammatory reaction and delayed wound healing process up to the 3rd month as evidenced by persistence of α-SMA stained cells [22–26].

Inflammation is a fundamental process in corneal wound healing. The infiltrating inflammatory cells and cytokines activate keratocytes differentiation into fibroblasts and myofibroblasts [22–26]. They migrate and accumulate in the provisional matrix of the wound site and secrete and deposit collagen [27, 28]. Severe inflammation might overwhelm the antiangiogenic mechanism of cornea and might give rise to a secondary ingrowth of blood vessels from the limbus into the central cornea [18, 29, 30]. Prolonged myofibroblasts activation and ongoing deposition of repair matrix would cause corneal scarring and opacification [31]. These two physiologic conditions may complicate corneal wound healing resulting to poor vision. With tissue engineered CG matrix providing the 3-dimensional porous scaffold and appropriate microenvironment to promote more fibroblast and myofibroblast repopulation, the healing processes are modified and optimized [9–11].

During the degradation of CG matrix, the patterns of cell migration and proliferation changes and the coexisting inflammation activates more fibroblasts and myofibroblasts. These keep depositing multiple elements of extracellular matrix to increase the corneal thickness [32]. By remodeling the healing stroma, and replacing the disorganized repair matrix with regular corneal extracellular matrix, a better transparency can be achieved [33, 34]. In addition, the number and the diameter of new vessels slowly decreases as the remodeling progresses [35].

In our study, the complete re-epithelization of the cornea wound on ungrafted eyes was achieved on day 14 but stroma remodeling was finished on day 28. In the grafted eyes, these results were observed on day 60 and day 90, respectively. These demonstrated that the wound healing proceeded from a proliferation phase to a stromal remodeling stage. Utsunomiya et al. in 2014 also observed the wound healing process after corneal stromal thinning shifted from an acute wound healing phase to a remodeling phase by anterior segment optical coherence tomography (OCT) [36]. The time to complete corneal wound healing in grafted eyes was influenced by the process and timing of CG matrix degradation. As a result, the intensity of corneal inflammation induced by CG matrix combined with the duration of CG degradation influenced the degree of stromal thickening, scarring and neovascularization.

CG matrix for repair of corneal thinning must be strong and implantable to maintain the corneal shape and curvature at the early stage. Additionally, the material should be able to maintain the 3-dimensional structure to support cell adhesion, migration and turnover into host extracellular matrix over time. In this study, we used soft contact lens and lateral tarsorrhaphy to anchor the CG matrix to corneal wound without further invasive procedures. This procedure minimizes disturbance of epithelium and maintains corneal shape without suturing, thereby avoids stimulation of an aggressive wound healing response. Though the main purpose of contact lens and tarsorrhaphy was to keep the CG graft in situ, they both also exerted certain pressure and compressed the CG matrix making it less prone to deformation. The CG matrix in our study was constructed by blending type I collagen with chondroitin 6-sulfate, with glutaraldehyde crosslinking. Compression test indicated that the matrix, upon compression by external force, becomes more rigid and becomes more difficult to deform [10]. In previous study, 2 mm thickness of collagen/C-6-S copolymer was used to repair conjunctival defect and the implantation reduced contraction and promoted the formation of a nearly normal subconjunctival stroma. The matrix was almost completely degraded on day 28 [9–11]. Therefore, 2 mm thickness collagen matrix with compression molding was used to resist the external forces by tarsorrhaphy and contact lens as well as enzymatic degradation. The majority of degradation of the CG matrix occurred in the first 4 postoperative weeks. Previous studies reported degradation rates of different collagen scaffolds to be 4 to 5 weeks, similar to what we observed in our study [37–39]. Based on the histopathologic findings of this study (Fig. 3), the 3-dimensional scaffold was maintained to a certain degree without collapsing, and this allows for cell migration and proliferation to happen. At the early stage, CG matrix maintains the good crosslinking structure to maintain the original porous structure without collapsing and result in the predictable randomized collagen deposition pattern. At the later stage, degrading CG matrix became less rigid and was flattened in a more parallel way by the pressure of tarsorrhaphy on it and resulted in better corneal clarity in the grafted group than expected.

In this study, CG matrix significantly increased the thickness of the healed cornea compared with ungrafted ones on day 90 (*P* < 0.001). From histological findings, the increased thickness resulted from additional lamella of new collagen deposition. Moreover, pentacam successfully measured the true central corneal thickness of the grafted eyes due to the improved clarity of the cornea. These results demonstrated that the new collagen ingrowth can undergo further remodeling to increase the corneal transparency. Although the corneas of grafted eyes were relatively hazy compared with the ungrafted ones, CG matrix was successful as a 3-dimensional temporary scaffold for corneal regeneration to regain the corneal thickness [39]. Corneal transparency greatly depends on the organization of the type I collagen fibrils, especially their diameter and regular lamellae organization [21]. While the slow degradation of CG matrix and inflammation induced by CG matrix lead to thicker cornea, both also resulted in corneal haziness. Therefore, finding the balance between corneal thickening and corneal haziness induced by CG matrix must be overcome. The limitation of this work is that corneal healing response from injury differs based on the nature of the insults such as chemical burn [40].

This study showed the healing effect of CG matrix on corneal thinning by injury. In the future, CG matrix may be designed with a different degradation rate so as to optimize stromal regeneration, or with a different porous size, diameter and arrangement to better regulate the assembly of cornea fibrils and the organization of the extracellular matrix to maintain corneal transparency [41, 42].

## Conclusion

Corneal perforation directly relates to the thickness of corneal stroma under the condition of corneal epithelial defect. Even though corneal scar formation and vessels ingrowth cause opacity of the cornea, less chance of corneal perforation is noted clinically. The 3-dimensional collagen-glycosaminoglycan scaffold offers a new microenvironment for cell migration, proliferation and differentiation but we still need to do more research to decrease the inflammation by modifying the degradation rate and create more parallel structure for corneal wound healing.

## Abbreviations

3D: three-dimensional
CG: collagen-glycosaminoglycan
DLK: deep lamellar keratectomy
OCT: optical coherence tomography
PMNs: polymorphonuclear neutrophils
α-SMA: alpha-smooth muscle actin
